# Comparing the Associations of Internet Addiction and Internet Gaming Disorder With Psychopathological Symptoms: Cross-Sectional Study of Three Independent Adolescent Samples

**DOI:** 10.2196/82414

**Published:** 2026-02-03

**Authors:** Ying-ying Li, A-qian Hu, Ling-li Yi, Zi-xin Mao, Qiu-yue Lü, Juan Wang, Wei Wei, Yue-qi Huang, Shu Huang, Wen-jing Dai, Meng-xuan Qiao, Jia-jun Xu, Qiang Wang, Xiao-jing Li, Fu-gang Luo, Wei Deng, Yu-zheng Hu, Tao Li, Wan-jun Guo

**Affiliations:** 1 Affiliated Mental Health Center & Hangzhou Seventh People's Hospital, School of Brain Science and Brain Medicine, Zhejiang University School of Medicine, 305 Tiamnyshan Rd, Hangzhou, Zhejiang, 310007, China, 86 18980602250; 2 MOE Key Lab for Neuroinformation, The Clinical Hospital of Chengdu Brain Science Institute, University of Electronic Science and Technology of China, Chengdu, Sichuan, China; 3 Mental Health Center and Psychiatric Laboratory, State Key Laboratory of Biotherapy, West China Hospital of Sichuan University, Chengdu, Sichuan, China; 4 Department of Psychiatry, Yichang Mental Health Center, Hubei, China; 5 School of Mental Health, Wenzhou Medical University, 325035, Wenzhou, China; 6 Department of Psychology and Behavioral Science, Zhejiang University, Hangzhou, 148 Tianmushan Road, Xihu District, Hangzhou, China; 7 Liangzhu Laboratory, MOE Frontier Science Center for Brain Science and Brain-Machine Integration, State Key Laboratory of Brain-Machine Intelligence, Zhejiang University, Hangzhou, 311121, China

**Keywords:** adolescent, attention deficit, hyperactivity, internet addiction, internet gaming disorder, psychopathology

## Abstract

**Background:**

Both internet gaming disorder (IGD) and internet addiction (IA) have been associated with diverse psychopathological symptoms. However, how the 2 conditions relate to each other and which is more strongly associated with psychopathology remain unclear.

**Objective:**

This study aimed to examine the association between IGD and IA and compare the strength of their associations with various types of psychopathological symptoms.

**Methods:**

This cross-sectional study surveyed 3 independent samples of Chinese adolescents: the first sample (S1) comprised 8194 first-year undergraduates at a comprehensive university in Chengdu, the second sample (S2) comprised 1720 students from a high school in Hangzhou, and the third sample (S3) comprised 551 inpatients aged 13 to 19 years recruited from 2 tertiary psychiatric hospitals in Hangzhou and Chengdu. IGD was defined as a score of 22 or more on the Internet Gaming Disorder Scale–Short Form (IGDS9-SF), whereas IA was defined as a score of 50 or more on Young’s 20-item Internet Addiction Test (IAT-20). Symptoms of depression, anxiety, psychoticism, paranoid ideation, and attention-deficit or hyperactivity were assessed using internationally validated scales including 9-item the Patient Health Questionnaire, 7-item Generalized Anxiety Disorder, psychoticism and paranoid ideation subscales of the Symptom Checklist 90 (absent for S2), and Adult ADHD Self-Report Scale (absent for S1), through online surveys in S1 (October 2020) and S3 (January 2022 to February 2025) and via an offline survey in S2 (March 2024).

**Results:**

The prevalence estimates (95% CI) of IGD were 4.8% (4.3%‐5.2%) in S1, 15.8% (14.0%‐17.5%) in S2, and 32.3% (28.4%‐36.2%) in S3, whereas prevalence estimates (95% CI) of IA were consistently higher across samples, ranging from 7.3% (6.8%‐7.9%) in S1 and 18.8% (17.0%‐20.6%) in S2 to 45.9% (41.8%‐50.1%) in S3. The IGDS9-SF and the IAT-20 were moderately correlated (Pearson *r*=0.51‐0.57; all *P*<.001) and were associated with the severity of most psychopathological symptom domains, with consistently stronger associations observed for IAT-20 scores. In multivariate models including all psychopathological symptoms as independent variables, the coefficients of determination (*R*², 95% CIs) were consistently higher for the IAT-20 than for the IGDS9-SF in S1 (0.33, 0.30‐0.35 vs 0.13, 0.11‐0.16) and S2 (0.44, 0.39‐0.49 vs 0.23, 0.18‐0.27), with a similar but nonsignificant pattern observed in S3 (0.13, 0.06‐0.26 vs 0.06, 0.03‐0.16). Post hoc analyses indicated that psychopathological symptoms were generally more severe in individuals with IA, either alone or comorbid with IGD, than in those with IGD only.

**Conclusions:**

This study provides additional evidence that IGD and IA are distinct yet interrelated constructs, and further demonstrates that IA consistently exhibits stronger associations with the severity of psychopathological symptoms than IGD. These findings underscore the importance of recognizing and addressing compulsive and problematic online behaviors that extend beyond gaming, highlighting the need to refine diagnostic frameworks and prioritize targeted clinical interventions.

## Introduction

In 2023, approximately 5.4 billion people were using the internet [1], many of whom were teenagers and young adults. These groups are at a critical stage of growth and development, which can be endangered by excessive or inappropriate use of the internet, potentially leading to internet gaming disorder (IGD) and internet addiction (IA) [2,3]. Recent systematic reviews and meta-analyses indicate that IA and IGD are increasingly prevalent worldwide, particularly among adolescents and young adults, and are associated with a growing burden of mental health and functional impairments [4,5].

IGD has been proposed in the fifth edition of the *Diagnostic and Statistical Manual of Mental Disorders* as a condition warranting further study [6], which specifies 9 dimensions of preoccupation, withdrawal, tolerance, unsuccessful attempts to stop or limit gaming, loss of interest in previous activities, continued use despite harm, avoidance, deception, and harm. The 11th edition of the *International Classification of Diseases, Eleventh Revision* (*ICD-11*) has defined gaming disorder, including predominantly online gaming, emphasizing impaired control, increasing priority given to gaming over other activities, and continued gaming despite negative consequences [7]. Both definitions imply significant social dysfunction arising from excessive online gaming. Studies based on one of the most widely used validated instruments for assessing internet gaming disorder that was proposed by a meta-analysis for screening internet gaming disorder, the Internet Gaming Disorder Scale–Short Form (IGDS9-SF) [8], suggest that the condition affects 9.3%-18.2% of adolescents in various countries [9,10]. Recent epidemiological and longitudinal studies have consistently shown that IGD in adolescents is associated with a wide range of adverse outcomes, including depressive and anxiety symptoms, attention-deficit or hyperactivity problems, aggression, sleep disturbances, poorer quality of life, and suicidality [11-13].

In contrast to IGD, IA has yet to be recognized as a bona fide disorder in the *Diagnostic and Statistical Manual* or other consensus guidelines [14]. It is typically defined as a behavioral addiction characterized by impaired control over internet use or online behaviors that results in clinically significant distress and functional impairment [15]. Studies using the most widely used instrument for assessing IA, the 20-item Young’s Internet Addiction Test (IAT-20) [16], suggest that up to 18% of adolescents may suffer from moderate IA and up to 1.5% may suffer from severe IA [17]. Studies based on the IAT-20 have linked IA to diverse psychopathological symptoms including depression, anxiety, obsessive-compulsive disorder, and suicidal ideation [18,19].

How IA and IGD relate to each other is unclear, with studies proposing that they are one and the same, that they are entirely separate from each other, or that one is a subtype of the other [20,21]. Although recent work has begun to assess generalized problematic internet use, gaming-specific problems, and other online behaviors within the same samples, highlighting both shared risk factors (eg, negative affect, maladaptive coping) and partially distinct symptom profiles across these conditions [22,23], their associations with psychopathological symptoms have not been systematically compared between IGD and IA within the same samples. Therefore, this study employed internationally validated instruments to assess IA and IGD in 3 adolescent samples from distinct settings in China, with the goal of examining the association between the 2 conditions and determining which is more strongly related to psychopathological symptom severity.

## Methods

### Participants

This study involved participants from 3 independent samples. Sample 1 (S1) comprised first-year undergraduates enrolled in 2020 at Sichuan University, a large comprehensive university in southwestern China that recruits students from all provincial-level administrative regions nationwide. As part of the Development of Psychological Health Assessment and Crisis Alarm and Intervention System project, all freshmen (n=9409) were invited to complete an online, self-administered psychosomatic health questionnaire via the university’s Online Psychosomatic Health Survey in October 2020. This study used the data from 8125 freshmen who provided valid responses to the Online Psychosomatic Health Survey, representing a response rate of 86.4%. Sample 2 (S2) comprised 1720 respondents (response rate: 78.2%) out of 2200 students invited to participate in a school-wide mental health survey at a high school in Hangzhou, China, conducted in March 2024 using paper-based questionnaires. Sample 3 (S3) comprised 551 respondents (response rate: 86.8%) out of 635 psychiatric inpatients aged 13‐19 years recruited from 2 psychiatric hospitals in Hangzhou and Chengdu, China, to complete online self-report scales of psychiatric symptoms between January 2022 and February 2025.

Individuals were excluded from the study if they were younger than 13 or older than 19 years, if they did not complete all questionnaire items, if their responses seemed unreliable (eg, endorsing the same symptom severity across all items), or if they used the same personal identification number as another participant. Undergraduates were excluded if they failed to submit their questionnaires properly through the online system or submitted them after the designated deadline.

### Measurements

The questionnaire packets contained queries on sociodemographic background along with various internationally validated assessment scales, including the IGDS9-SF, the IAT-20, the Patient Health Questionnaire-9 (PHQ-9), Generalized Anxiety Disorder-7 (GAD-7), psychoticism and paranoid Ideation Subscales of the Symptom Checklist 90 (administered only for S1 and S3), and Adult ADHD Self-Report Scale (ASRS; administered only for S2 and S3). These instruments were used to assess IGD, IA, and 5 domains of psychopathology (depression, anxiety, psychoticism, paranoid ideation, and attention-deficit or hyperactivity symptoms), respectively.

#### Internet Gaming Disorder Scale–Short Form

The IGDS9-SF [8] assesses the frequency of symptoms of IGD during the previous 12 months. The symptoms correspond to the diagnostic criteria of the 5th edition of the *Diagnostic and Statistical Manual* and the features defined by the 11th edition of the *International Classification of Diseases*. The IGDS9-SF is one of the few questionnaires assessing IGD that includes the criterion of “continued internet use despite harm.” Participants respond to each of the 9 items on a 5-point Likert scale from 1 (“rarely”) to 5 (“always”), yielding a total score ranging from 0 to 45. Scores ≥22 were defined as indicative of IGD [24]. Cronbach α in all 3 samples of this study ranged from 0.89 to 0.93.

#### Twenty-Item Young’s Internet Addiction Test

The IAT-20 [25] is the most frequently used self-report measure of problematic internet use [26]. Participants respond to each of the 20 items on a 5-point Likert scale from 1 (“rarely”) to 5 (“always”) or by entering the value 0 (“not applicable”), yielding a total score from 0 to 100. Scores ≥50 were considered indicative of IA [27]. In this study, Cronbach α across the 3 samples ranged from 0.93 to 0.94.

#### Nine-Item Patient Health Questionnaire

The PHQ-9 assesses the severity of depressive symptoms over the past 2 weeks. Participants respond to each of the 9 items using a 4-point scale ranging from 0 (“not at all”) to 3 (“nearly every day”), yielding a total score range of 0‐27. Higher scores indicate greater depressive symptom severity. The scale has demonstrated satisfactory psychometric properties among Chinese populations [28]. In this study, Cronbach α across the 3 samples ranged from 0.86 to 0.91.

#### Seven-Item Generalized Anxiety Disorder

The GAD-7 [29] assesses the severity of anxiety symptoms during the previous 2 weeks. Participants respond to each of 7 items on a 4-point scale from 0 (“not at all”) to 3 (“nearly every day”), yielding a total score range of 0-21. Higher scores indicate greater anxiety symptom severity. The Chinese version has demonstrated satisfactory validity and reliability. Cronbach α in all 3 samples of this study ranged from 0.90 to 0.93.

#### Psychoticism and Paranoid Ideation Subscales of the Symptom Checklist-90

The severity of psychoticism and paranoid ideation was assessed using the corresponding subscales of the Symptom Checklist-90. Participants respond to each of the 10 items on the psychoticism subscale or 6 items on the paranoid ideation subscale using a 5-point Likert scale ranging from 0 (“not at all”) to 4 (“extremely”). The SCL-90 has demonstrated robust validity and reliability in Chinese populations [30]. These subscales were administered only in S1 (undergraduates) and S3 (inpatients). Cronbach α was 0.83 (S1) and 0.79 (S3) for psychoticism and 0.87 (S1) and 0.84 (S3) for paranoid ideation.

#### Adult ADHD Self-Report Scale

The ASRS, widely used clinically to screen adults for attention-deficit or hyperactivity disorder, is based on the 18 criteria in the “TR” revision of the 4th edition of the *Diagnostic and Statistical Manual of Mental Disorders* [31]. It examines symptoms over the past 6 months. Participants respond to 18 items on a Likert scale from 0 (“never”) to 4 (“very often”), giving a total score from 0 to 72. Higher scores indicate greater ADHD symptom severity. The Chinese version has demonstrated both reliability and validity in young adults in Taiwan [32]. This survey was administered only in S2 (high school students) and S3 (inpatients). Cronbach α was 0.93 in S2 and 0.91 in S3.

### Analysis

The data were analyzed using SPSS 27.0 (IBM) and R (version 4.4.3; R Core Team). Descriptive statistics, correlation analyses, independent-samples *t* tests, ANOVA, and post hoc comparisons were conducted in SPSS, whereas regression-related analyses, including the computation of confidence intervals for *R*² (not available in SPSS), were performed in R. The significance of univariate associations was assessed using, as appropriate, Pearson correlation coefficients (*r*), 2-tailed *t* tests, chi-square tests, or ANOVA. ANOVA results were adjusted using Tukey honest significant difference procedure to control for multiple comparisons. Potential relationships among variables were explored using multiple linear and logistic regression. To control for the confounding effects of demographics, we used standardized residuals (ZREs) of psychopathology scores adjusted for age and sex in regression and post hoc analyses; descriptive statistics were based on raw scores to facilitate comparability with prior studies. To evaluate whether multicollinearity among independent variables (eg, *r*>0.7 between the PHQ-9 and the GAD-7) affected the reliability of our regression models, we obtained tolerance and variance inflation factors before multivariate regression analyses. Where appropriate, associations were expressed as odds ratios (ORs) with corresponding 95% CIs. The results were considered statistically significant at a 2-tailed *P*<.05. The missing data in S2 were handled using complete-case analysis without multiple imputation after testing for missing completely at random, as item-level missingness was minimal (0.2%‐3.0% per item). S1 and S3 used forced-response online questionnaires, resulting in no item-level missingness. The ASRS data in S3 were available only for 241 participants because the scale was introduced midway through the study. Accordingly, analyses involving the ASRS in S3 included only these 241 participants.

### Ethical Considerations

The study procedures were carried out in accordance with the Declaration of Helsinki, and the study was conducted and reported in accordance with the Journal Article Reporting Standards [33]. The study was approved by the Ethics Committee of West China Hospital, Sichuan University (approval No. 2016‐171), and the Ethics Committee of Hangzhou Seventh People’s Hospital (approval No. 2023‐064). All S1 participants provided electronic informed consent; for S2 and S3, both participants and their parents received detailed study information and provided written informed consent. All data were anonymized and stored securely on password-protected servers, and only aggregated results were reported. Participants did not receive any financial or material compensation for their participation in this study.

## Results

### Demographics

Male participants constituted a slight majority in S1 (n=8194, 54.5%), were slightly underrepresented in S2 (n=1720, 43.8%), and were substantially underrepresented in S3 (n=551, 26.0%). Mean ages (95% CI) in years were 18.1 (18.1-18.1) among undergraduates, 17.3 (17.0-17.6) among high school students, and 15.6 (15.4-15.7) among inpatients.

### Measurement Scores of IGD and IA and Their Correlation With Demographics

The mean IGDS9-SF score (95% CI) in S1 was 11.8 (11.7-12.0), which was significantly lower than in S2 (15.1, 14.8-15.4), and S2 was significantly lower than S3 (18.8, 17.8-19.7). Similar trends were observed across samples for IA. The mean IAT-20 score (95% CI) in S1 was 31.7 (31.5-32.0), which was significantly lower than in S2 (32.4, 31.5-33.3), and S2 was significantly lower than S3 (47.7, 45.5-49.9; Table 1).

**Table 1.** Mean scores of measurements of internet gaming disorder (IGD), internet addiction (IA), and psychopathology, and the Pearson correlation matrix among demographic variables and these measures, across the 3 samples^a^.

Measure	Scores, mean (95% CI)	Pearson correlation coefficients (*r*) among demographic variables and measures of IGD, IA, and psychopathology								
		Age	Gender	IAT-20^b^	IGDS9-SF^c^	PHQ-9^d^	GAD-7^e^	SCL90-psychoticism^f^	SCL90–paranoid ideation^g^	ASRS^h^
Sample 1 (n=8194)										
IGDS9-SF	11.8 (11.7-12.0)	0.02	–0.28^i^	0.51^i^	1.00	0.27^i^	0.24^i^	0.32^i^	0.27^i^	—^j^
IAT-20	31.7 (31.5-32.0)	–0.02^k^	0.08^i^	1.00	0.51^i^	0.50^i^	0.46^i^	0.52^i^	0.48^i^	—
PHQ-9	3.0 (2.9-3.1)	–0.00	0.12^i^	0.50^i^	0.27^i^	1.00	0.77^i^	0.63^i^	0.56^i^	—
GAD-7	2.0 (1.9-2.1)	–0.00	0.10^i^	0.46^i^	0.24^i^	0.77^i^	1.00	0.61^i^	0.56^i^	—
SCL90-psychoticism	1.2 (1.2-1.3)	–0.01	0.03^l^	0.52^i^	0.32^i^	0.63^i^	0.61^i^	1.00	0.80^i^	—
SCL90–paranoid ideation	1.3 (1.2-1.3)	–0.02	0.06^i^	0.48^i^	0.27^i^	0.56^i^	0.56^i^	0.80^i^	1.00	—
Sample 2 (n=1720)										
IGDS9-SF	15.1 (14.8-15.4)	–0.02	–0.24^i^	0.55^i^	1.00	0.35^i^	0.30^i^	—	—	0.41^i^
IAT-20	32.4 (31.5-33.3)	0.05^k^	0.09^i^	1.00	0.55^i^	0.54^i^	0.48^i^	—	—	0.62^i^
PHQ-9	6.3 (6.0-6.5)	0.03	0.11^i^	0.54^i^	0.35^i^	1.00	0.69^i^	—	—	0.55^i^
GAD-7	4.1 (3.9-4.2)	0.02	0.07^l^	0.48^i^	0.30^i^	0.69^i^	1.00	—	—	0.55^i^
ASRS	22.8 (22.3-23.3)	–0.01	0.05^k^	0.62^i^	0.41^i^	0.55^i^	0.55^i^	—	—	1.00
Sample 3										
IGDS9-SF (n=551)	18.8 (17.8-19.7)	–0.10^k^	–0.14^l^	0.57^i^	1.00	0.13	0.08	0.14^k^	0.16^k^	0.24^i^
IAT-20 (n=551)	47.7 (45.5-49.9)	–0.09^k^	0.03	1.00	0.57^i^	0.24^i^	0.16^k^	0.26^i^	0.18^l^	0.35^i^
PHQ-9 (n=551)	18.2 (17.4-19.1)	–0.16^i^	0.13^l^	0.24^i^	0.13	1.00	0.77^i^	0.67^i^	0.57^i^	0.57^i^
GAD-7 (n=551)	13.5 (12.9-14.1)	–0.14^l^	0.11^l^	0.16^k^	0.08	0.77^i^	1.00	0.69^i^	0.58^i^	0.53^i^
SCL90-psychoticism (n=551)	2.5 (2.3-2.6)	–0.18^i^	0.06	0.26^i^	0.14^k^	0.67^i^	0.69^i^	1.00	0.77^i^	0.67^i^
SCL90–paranoid ideation (n=551)	2.5 (2.3-2.7)	–0.17^i^	0.07	0.18^l^	0.16^k^	0.57^i^	0.58^i^	0.77^i^	1.00	0.59^i^
ASRS (n=241)^m^	54.0 (51.8-56.2)	–0.17^l^	0.12	0.35^i^	0.24^i^	0.57^i^	0.53^i^	0.67^i^	0.59^i^	1.00

a Sample 1 comprises undergraduate freshmen enrolled at Sichuan University. Sample 2 comprises students recruited from a high school in Hangzhou. Sample 3 comprises inpatients aged 13‐19 years recruited from 2 tertiary mental health centers in Hangzhou and Chengdu. The Internet Gaming Disorder Scale–Short Form, 20-item Young’s Internet Addiction Test, Patient Health Questionnaire-9, and Generalized Anxiety Disorder-7 were administered in all 3 samples; the SCL-90 Psychoticism and Paranoid Ideation subscales were administered only in S1 and S3, and the Adult ADHD Self-Report Scale was administered only in S2 and S3.

b IAT-20: 20-item Young’s Internet Addiction Test.

c IGDS9-SF: Internet Gaming Disorder Scale–Short Form.

d PHQ-9: 9-item Patient Health Questionnaire.

e GAD-7: 7-item Generalized Anxiety Disorder.

f SCL90-psychoticism: subscale of the Symptom Checklist-90 to measure psychoticism.

g SCL90–paranoid ideation: subscale of the Symptom Checklist-90 to measure paranoid ideation.

h ASRS: Adult ADHD Self-Report Scale.

i *P*<.001.

j Not available.

k *P*<.05.

l *P*<.01.

m The Adult ADHD Self-Report Scale data in sample 3 were available only for 241 participants because the scale was introduced midway through the study.

IGDS9-SF and IAT-20 scores showed either statistically significant but modest correlations with age or nonsignificant associations across all 3 samples (*r*=–0.10 to 0.10; Table 1). The scores of the IGDS9-SF were significantly higher in male than in female participants in all 3 samples (*t*_8192_=26.2, *P*<.001 in S1; *t*_1718_=9.92,* P*<.001 in S2; and *t*_549_=3.18*, P=.*002 in S3). Conversely, the scores of the IAT-20 were significantly higher in female than in male participants in S1 (*t*_8192_=–7.00; *P*<.001) and S2 (*t*_1718_=–3.67;* P*<.001), whereas no significant sex differences were observed in S3 (*t*_549_=0.61;* P*=.55).

### Prevalence Rates of IGD and IA

The prevalence (95% CI) of IGD in S1, based on the predefined cutoff (IGDS9-SF ≥22), was 4.8% (4.3%-5.2%), which was significantly lower than in S2 (15.8%, 14.0%-17.5%), and in turn significantly lower than in S3 (32.3%, 28.4%-36.2%). The prevalence (95% CI) of IA in S1, based on the predefined cutoff (IAT-20 ≥50), was 7.3% (6.8%-7.9%), which was significantly lower than in S2 (18.8%, 17.0%-20.6%), and in turn was significantly lower than that in S3 (45.9%, 41.8%-50.1%).

In other words, in S1 (n=8194), most undergraduates (n=7408, 90.4%, 95% CI 89.8%-91.1%) had neither IGD nor IA, whereas small proportions (n=184, 2.24%, 95% CI 1.94%-2.59%) had IGD only, IA only (n=396, 4.83%, 95% CI 4.38%-5.32%), or both (n=206, 2.51%, 95% CI 2.19%-2.88%). In S2 (n=1720), a smaller majority (n=1287, 74.83%, 95% CI 72.75%-76.83%) of the high school students had neither disorder, with correspondingly higher prevalence of only IGD (n=109, 6.34%, 95% CI 5.27%-7.59%), only IA (n=163, 9.48%, 95% CI 8.17%-10.94%), and both (n=161, 9.36%, 95% CI 8.06%-10.83%). In contrast, in S3 (n=551), a slight majority of the inpatients had one or both disorders: only 253 (45.92%, 95% CI 41.71%-50.18%) had neither disorder, whereas 45 (8.17%, 95% CI 6.08%-10.86%) had IGD only, 120 (21.78%, 95% CI 18.45%-25.51%) had IA only, and 133 (24.14%, 95% CI 20.67%-27.98%) had both (Table 1).

### Associations of Measurements and Prevalence Between IGD and IA

Univariate analyses indicated that IGDS9-SF and IAT-20 scores were moderately correlated in all 3 samples (*r*=0.51, *P*<.001 in S1; *r*=0.55, *P*<.001 in S2; and *r*=0.51, *P*<.001 in S3). These correlations remained significant after controlling for sex and age (Table 1). Similarly, multivariable analyses controlling for sex and age indicated that the odds of IA were significantly higher among participants with IGD in all 3 samples, with adjusted ORs (95% CI) of 29.6 (23.2-37.9) in S1, 14.1 (10.3-19.6) in S2, and 6.5 (4.3-9.9) in S3.

### Scores of Psychopathological Symptoms

The mean PHQ-9 score (95% CI) in S1 was 3.0 (2.9-3.1), which was significantly lower than in S2 (6.3, CI 6.0-6.5) and in turn significantly lower than in S3 (18.2, 17.4-19.1). Similarly, the mean GAD-7 score (95% CI) in S1 was 2.0 (1.9-2.1), which was significantly lower than in S2 (4.1, 3.9-4.2), and in turn was significantly lower than in S3 (13.5, 12.9-14.1). The mean SCL-90 psychoticism subscale score (95% CI) in S1 was 1.2 (1.2-1.3), which was significantly lower than in S3 (2.5, 2.3-2.6). Similarly, the mean SCL-90 paranoid ideation subscale score (95% CI) in S1 was 1.3 (1.2-1.3), which was significantly lower than that in S3 (2.5, 2.3-2.7). The mean ASRS score (95% CI) in S2 was 22.8 (22.3-23.3), which was significantly lower than that in S3 (54.0, 51.8-56.2).

Psychopathology severity was not associated with age in S1 or S2 but showed small negative correlations with age in S3 (*r*=–0.09 to –0.18). All measured types of psychopathological symptoms in S1 and S2 were significantly more severe in female than in male participants, whereas only depression (PHQ-9) and anxiety (GAD-7) exhibited this sex difference in S3 (Table 1).

### Associations of IGD or IA With Severity of Psychopathological Symptoms

To examine whether IGD or IA was associated with psychopathology, we performed pairwise comparisons between participants with and without IGD and between participants with and without IA; these analyses revealed statistically significant differences across all psychopathological variables in each sample. Notably, participants with IA exhibited significantly higher dimensional psychopathology scores than those with IGD on the PHQ-9, GAD-7, and the SCL-90 paranoid ideation subscale in S1. A similar trend of differences was observed in S2 and S3, although the differences did not reach statistical significance (Table 2).

**Table 2.** Mean scores (95% CI) of measurements for internet gaming disorder (IGD), internet addiction (IA), and other psychopathologies among all participants, those with IGD and IA, and those without IGD or IA, across the 3 samples^a^.

Sample and psychopathologies	Scores among participants with IGD^b^, mean (95% CI)	Scores among participants with IA^c^, mean (95% CI)	Scores among participants without IGD^d^, mean (95% CI)	Scores among participants without IA^e^, mean (95% CI)
Sample 1				
IGDS9-SF^f^	25.2 (24.7-25.7)	17.6 (17.0-18.2)	11.2 (11.1-11.2)	11.4 (11.3-11.5)
IAT-20^g^	50.8 (49.3-52.3)	58.2 (57.5-58.8)	30.8 (30.5-31.0)	29.6 (29.4-29.8)
PHQ-9^h^	6.0 (5.5-6.5)	7.2 (6.9-7.6)	2.8 (2.8-2.9)	2.7 (2.6-2.7)
GAD-7^i^	4.6 (4.1-5.1)	5.6 (5.3-6.0)	1.9 (1.9-2.0)	1.8 (1.7-1.8)
SCL90-psychoticism^j^	1.6 (1.5-1.7)	1.7 (1.6-1.7)	1.2 (1.2-1.2)	1.2 (1.2-1.2)
SCL90–paranoid ideation^k^	1.7 (1.6-1.7)	1.8 (1.7-1.8)	1.3 (1.3-1.3)	1.2 (1.2-1.3)
Sample 2				
IGDS9-SF	26.5 (25.9-27.0)	21.3 (20.4-22.2)	13.0 (12.8-13.2)	13.6 (13.4-13.8)
IAT-20	53.4 (51.3-55.4)	62.8 (61.8-63.9)	28.4 (27.5-29.4)	32.4 (31.9-32.8)
PHQ-9	9.8 (9.1-10.5)	10.6 (10.0-11.2)	5.7 (5.4-5.9)	5.2 (5.0-5.4)
GAD-7	6.8 (6.1-7.5)	7.4 (6.9-7.9)	3.5 (3.3-3.8)	3.2 (3.1-3.3)
ASRS^l^	31.9 (30.4-33.5)	33.9 (32.7-35.0)	21.0 (20.3-21.6)	20.0 (19.4-20.6)
Sample 3				
IGDS9-SF	30.0 (29.0-30.9)	23.1 (21.9-24.4)	13.5 (13.0-13.9)	15.1 (14.3-15.8)
IAT-20	60.9 (58.1-63.8)	67.2 (65.6-68.8)	41.4 (39.4-43.5)	31.2 (29.7-32.6)
PHQ-9	17.9 (17.0-18.9)	18.9 (17.8-19.9)	18.4 (17.7-19.1)	17.7 (16.9-18.5)
GAD-7	13.2 (12.4-14.1)	14.0 (13.3-14.7)	13.6 (13.0-14.3)	13.1 (12.4-13.8)
SCL90-psychoticism	2.6 (2.4-2.7)	2.7 (2.6-2.9)	2.4 (2.3-2.5)	2.2 (2.1-2.3)
SCL90–paranoid ideation	2.7 (2.5-2.9)	2.8 (2.7-2.9)	2.4 (2.3-2.5)	2.3 (2.1-2.4)
ASRS^m^	56.5 (54.0-59.0)	56.7 (54.9-58.5)	52.4 (50.3-54.4)	50.0 (46.3-53.7)

a Internet gaming disorder was defined as a total score ≥22 on the IDSG9-SF, and internet addiction was defined as a total score ≥50 on Young’s 20-item Internet Addiction Test (IAT-20).

b Sample sizes for participants with internet gaming disorder: sample 1: n=393; sample 2: n=271; sample 3: n=178.

c Sample sizes for participants with internet addiction: sample 1: n=598; sample 2: n=323; sample 3: n=253.

d Sample sizes for participants without internet gaming disorder: sample 1: n=7801; sample 2: n=1,449; sample 3: n=373.

e Sample sizes for participants without internet addiction: sample 1: n=7596; sample 2: n=1397; sample 3: n=298.

f IDSG9-SF: Internet Gaming Disorder Scale–Short Form.

g IAT-20: 20-item Young’s Internet Addiction Test.

h PHQ-9: 9-item Patient Health Questionnaire.

i GAD-7: 7-item Generalized Anxiety Disorder.

j SCL90-psychoticism: subscale of the Symptom Checklist-90 to measure psychoticism.

k SCL90–paranoid ideation: subscale of the Symptom Checklist-90 to measure paranoid ideation.

l ASRS: Adult ADHD Self-Report Scale.

m For the ASRS in sample 3, there were 88 participants with IGD, 10 with IA, 59 without IGD, and 84 without IA.

Next, we examined whether IGDS9-SF and IAT-20 scores were associated with psychopathological symptom scores. In these analyses, the independent variables were the ZREs of psychopathological symptom scores, adjusted for age and sex; the dependent variable was the ZRE of the IGDS9-SF or IAT-20 score. Univariate models indicated that both IGDS9-SF and IAT-20 scores were significantly associated with all psychopathological symptom scores. These associations were significantly stronger for the IAT-20 than for the IGDS9-SF, as indicated by higher *R*² values, in S1 and S2. A similar trend was observed in S3, though most differences in associations did not reach statistical significance in this sample. Consistently, multivariate models, in which multicollinearity was acceptable (all tolerances >0.20, 95% CI 0.28‐0.53, and all variance inflation factors <5, 95% CI 1.55‐3.55), showed that psychopathological symptom scores were related to a larger proportion of changes in IAT-20 scores than IGDS9-SF scores in S1 (0.33, 95% CI 0.30-0.35 vs 0.13, 95% CI 0.11-0.16) and S2 (0.44, 95% CI 0.39-0.49 vs 0.23, 95% CI 0.18-0.27). A similar trend was observed in S3, although it did not reach statistical significance.

We also performed regression analyses in which IGD or IA status (present vs absent), defined using the prespecified IGDS9-SF and IAT-20 cutoff scores, served as the dependent variables, and the ZREs of psychopathological symptom scores served as the independent variables. In univariate models, both IGD and IA were significantly associated with all psychopathological symptom scores in S1 and S2, and with some of these symptom scores in S3. These associations were significantly stronger for IA than for IGD, as indicated by larger ORs, in S1 and S2. A similar trend was observed in S3, although most differences in ORs did not reach statistical significance in this sample. Multivariate analyses showed a similar pattern in which ORs were generally larger for IA than for IGD, particularly in S1 and S2, although most differences did not reach statistical significance.

Notably, in S2, IAT-20 scores showed a stronger univariate association with attention-deficit or hyperactivity symptom severity (ASRS scores) (*R*^2^, 95% CI; 0.39, 0.33-0.43) than with depression (0.28, 0.23-0.32) or anxiety (0.22, 0.18-0.26). IGDS9-SF scores (*R*^2^, 95% CI) also showed a stronger univariate association with attention deficit or hyperactivity symptom severity (0.19, 0.14-0.23) than with anxiety (0.10, 0.07-0.14). Similar trends were observed in S3, although they did not reach statistical significance. In the logistic regression analyses, univariate models showed that ASRS scores had the strongest associations with IGD and IA among all psychopathological measures in both S2 and S3, although differences in association strength, in terms of ORs, did not reach statistical significance. In multivariate models adjusting for the intercorrelation among other psychopathological measures, higher ASRS scores were independently associated with increased odds of IGD (OR, 95% CI; S2: 2.17, 1.80‐2.65; S3: 1.43, 0.99-2.09) and IA (S2: 3.18, 2.57‐3.96; S3: 1.75, 1.19-2.64). By contrast, most associations between other psychopathological symptoms and IGD or IA that were significant in univariate models were no longer statistically significant after multivariate adjustment (Table 3).

**Table 3.** Associations of Internet Gaming Disorder Scale–Short Form (IGDS9-SF), 20-item Young’s Internet Addiction Test (IAT-20), and identified internet gaming disorder (IGD) and internet addiction (IA) with the severities of other psychopathologies across three samples^a^.

Sample and psychopathology	IGDS9-SF^b^		IAT-20^b^		IGD		IA	
	R^2^ (95% CI) in univariate model	R^2^ (95% CI) in multivariate model^c^	R^2^ (95% CI) in univariate model	R^2^ (95% CI) in multivariate model^c^	OR^d^ (95% CI)	Adjusted OR^c^ (95% CI)	OR (95% CI)	Adjusted OR^c^ (95% CI)
Sample 1		0.13 (0.11-0.16)		0.33 (0.30-0.35)				
PHQ-9^b^^,^^e^	0.10 (0.08-0.12)		0.25 (0.23-0.27)		1.89 (1.76-2.04)	1.33 (1.16-1.53)	2.50 (2.33-2.69)	1.50 (1.34-1.69)
GAD-7^b^^,^^f^	0.08 (0.06-0.10)		0.21 (0.19-0.23)		1.76 (1.64-1.89)	1.09 (0.95-1.23)	2.23 (2.09-2.39)	1.22 (1.09-*1.35)*
SCL90-psychoticism^b^^,^^g^	0.12 (0.10-0.14)		0.27 (0.25-0.30)		1.87 (1.75-2.00)	1.24 (1.08-1.42)	2.40 (2.25-2.58)	1.54 (1.36-1.74)
SCL90–paranoid ideation^b^^,^^h^	0.09 (0.07-0.11)		0.23 (0.21-0.25)		1.88 (1.75-2.01)	1.32 (1.16-1.51)	2.21 (2.07-2.36)	1.16 (1.03-1.30)
Sample 2		0.23 (0.18-0.27)		0.44 (0.39-0.49)				
PHQ-9^b^	0.15 (0.11-0.19)		0.28 (0.23-0.32)		2.20 (1.93-2.52)	1.56 (1.28-1.91)	3.08 (2.66-3.58)	1.89 (1.54-2.31)
GAD-7^b^	*0.10 (0.07-0.14)*		0.22 (0.18-0.26)		1.90 (1.68-2.15)	1.02 (0.84-1.23)	2.47 (2.17-2.83)	1.09 (0.90-1.31)
ASRS^b^^,^^i^	0.19 (0.14-0.23)		0.39 (0.33-0.43)		2.85 (2.43-3.38)	2.17 (1.80-2.65)	4.60 (3.80-5.64)	3.18 (2.57-3.96)
Sample 3		0.06 (0.03-0.16)		0.13 (0.06-0.26)				
PHQ-9^b^	0.00 (0.00-0.01)		0.01 (0.00-0.03)		0.93 (0.78-1.12)	0.92 (0.60-1.41)	1.16 (0.98-1.37)	1.21 (0.79-1.85)
GAD-7^b^	0.00 (0.00-0.00)		0.00 (0.00-0.02)		0.93 (0.78-1.11)	0.89 (0.57-1.40)	1.13 (0.95-1.34)	0.85 (0.54-1.33)
SCL90-psychoticism^b^	0.02 (0.00-0.05)		0.10 (0.06-0.16)		1.16 (0.97-1.40)	0.98 (0.59-1.63)	1.71 (1.43-2.06)	1.17 (0.69-1.99)
SCL90–paranoid ideation^b^	0.03 (0.01-0.07)		0.11 (0.06-0.16)		1.32 (1.09-1.59)	1.21 (0.78-1.87)	1.65 (1.37-1.99)	0.80 (0.50-1.24)
ASRS^b^	0.06 (0.01-0.14)		0.11 (0.02-0.22)		1.40 (1.07-1.85)	1.43 (0.99-2.09)	1.73 (1.30-2.36)	1.75 (1.19-2.64)

a IGD was defined based on the total score of the IGDS9-SF, with a cutoff value of ≥21. IA was defined based on the total score of IAT-20, with a cutoff value of ≥50. *R*² represents the square of the correlation coefficient (ie, *r*).

b Standardized residuals (ZREs) adjusted for the confounding effects of age and sex of measurement scores were used as dependent or independent variables in the regression models.

c These multivariate models included ZREs of other psychopathological symptom measures as independent variables.

d ORs: odds ratios.

e PHQ-9: 9-item Patient Health Questionnaire.

f GAD-7: 7-item Generalized Anxiety Disorder.

g SCL90-psychoticism: subscale of the Symptom Checklist-90 to measure psychoticism.

h SCL90–paranoid ideation: subscale of the Symptom Checklist-90 to measure paranoid ideation.

i ASRS: Adult ADHD Self-Report Scale. The ASRS data in S3 were available for only 241 participants because the scale was added midway through the study.

**Figure 1.**
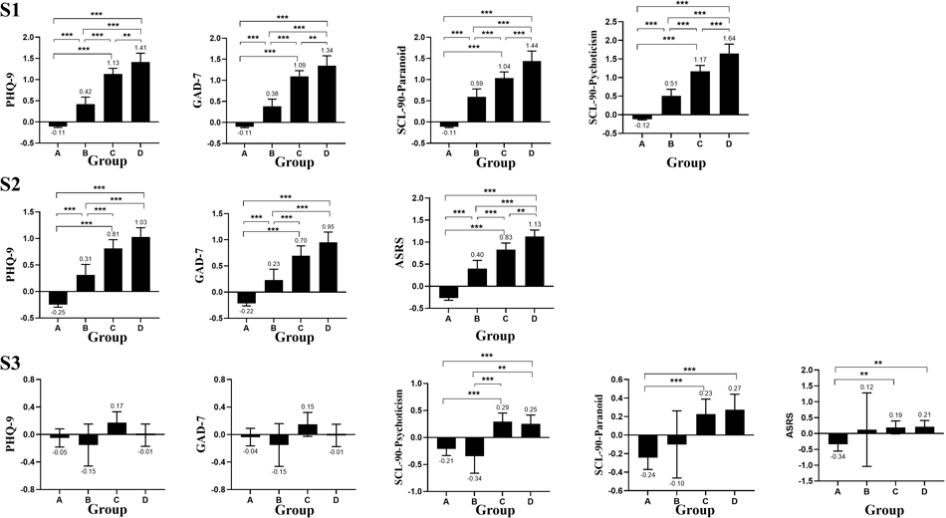
Post hoc comparisons of psychopathological symptom severity (*z*-standardized residuals adjusted for age and sex) among participants with neither internet gaming disorder (IGD) nor internet addiction (IA; group A), IGD only (group B), IA only (group C), or comorbid IGD and IA (group D) across 3 independent samples: S1 (first-year undergraduates at Sichuan University), S2 (high school students recruited in Hangzhou), and S3 (inpatients aged 13‐19 years recruited from 2 tertiary mental health centers in Hangzhou and Chengdu). ASRS: Adult ADHD Self-Report Scale; GAD-7: 7-item Generalized Anxiety Disorder; PHQ-9: 9-item Patient Health Questionnaire; SCL-90-paranoid: subscale of the Symptom Checklist-90 to measure paranoid ideation; SCL-90-psychotism: subscale of the Symptom Checklist-90 to measure psychoticism. **P<.01; ***P<.001.

Finally, we classified participants into 4 groups according to IGD and IA status: both IGD and IA, neither condition, IGD only, or IA only. ANOVA tests indicated that ZREs of all measured psychopathological symptoms differed significantly across the 4 groups in all 3 samples. Post hoc analyses in S1 revealed a significant stepwise increase in depressive, anxiety, psychoticism, and paranoid ideation symptom severity from participants with neither disorder to those with IGD only, then to those with IA only, and finally to those with both disorders. Similar trends were observed in S2 for the severity of depression, anxiety, and attention-deficit or hyperactivity, although the differences in depressive and anxiety symptom severity between the IA-only group and the comorbid group were not statistically significant. In S3, participants with IA (with or without comorbid IGD) tended to have higher psychopathological symptom scores than those with neither disorder nor IGD only, although some post hoc comparisons did not reach statistical significance (Figure 1).

## Discussion

### Findings

To our knowledge, this is the first study to examine a potential relationship between IGD and IA, as well as to compare their associations with a broad range of psychopathological symptoms within the same samples. In addition, we cross-validated the main findings across 3 independent samples. Across samples, we found that IGD and IA were distinct yet moderately correlated and that IA was more prevalent and more strongly associated with psychopathological symptom severity.

These key findings have important implications for clarifying the nosological relationship between IGD and IA. The moderate association we detected between the 2 disorders was similar to that reported between so-called “problematic internet use” and “problematic online gaming” [20], but it was weaker than the associations between depressive and anxiety symptoms observed in this study and in previous work in other Chinese samples [34,35]. These observations support considering IGD and IA as distinct yet correlated entities, rather than as a single entity or as a subtype of a broader construct [21]. Consistent with this interpretation, we identified participants who met the most widely accepted definition of IGD but not IA, and vice versa. In our sample, IA, whether occurring alone or comorbid with IGD, was associated with more severe psychopathological symptoms than IGD alone. Furthermore, our univariate correlation and multivariate regression analyses indicated that IA severity was consistently more strongly associated with psychopathological symptom severity than IGD severity. These findings suggest that compulsive, problematic online behaviors extending beyond gaming warrant greater attention. Although “gaming” is currently the only type of online activity codified in the *International Classification of Diseases* and listed as a condition for further study in the *Diagnostic and Statistical Manual of Mental Disorders, Fifth Edition* (*DSM-5*), other forms of online entertainment, such as social media and algorithm-driven short-video platforms, may pose comparable or even greater risks to mental health and may undermine psychological well-being, academic achievement, and family dynamics [36,37]. It may be more appropriate to refer to broader constructs such as “internet entertainment disorder” or “internet entertainment addiction” to better capture the spectrum of addictive behaviors related to internet use. These constructs may require further expansion or adaptation to reflect the growing use of immersive technologies, such as virtual or augmented reality and artificial intelligence, which may further facilitate addictive behaviors.

In our study, both IGD and IA were more strongly associated with symptoms of attention-deficit or hyperactivity symptoms than with depressive or anxiety symptoms, consistent with findings from a study of adults aged 20‐40 years in Taiwan [38]. These findings suggest that ADHD-related inattention and impulsivity, as indexed by ASRS scores, may partially account for the observed associations between IGD or IA and other psychopathological symptoms. Evidence from adolescents in several countries further indicates that attention-deficit or hyperactivity disorder frequently co-occurs with IGD and IA and may even predict their onset [39]. Core features of attention-deficit or hyperactivity, including impulsivity, inattention, heightened sensation seeking, and poor regulatory control, may increase the risk of problematic internet use [40-42]. More broadly, both disorders may be more strongly associated with externalizing symptoms characteristic of attention-deficit or hyperactivity than with internalizing symptoms, such as depression or anxiety, a pattern supported by recent meta-analytic evidence, although the pooled effect sizes were modest [43]. This closer association with externalizing symptoms may be related to the immediate gratification and heightened sensory stimulation inherent in gaming and other forms of online entertainment [44,45]. In contrast, internalizing symptoms may instead reflect maladaptive coping mechanisms for psychological distress [46,47].

We consider our data to be reliable because we assessed IGD and IA using widely validated, commonly used instruments and because we were able to replicate key associations across the 3 independent samples of adolescents. In addition, the differences in psychopathological symptoms observed across samples were consistent with expectations: hospitalized adolescents exhibited high levels of psychopathological symptoms similar to those reported in a study of Caucasian adolescents [48], and these levels were higher than those in high school students, which in turn were higher than those observed in undergraduates. Our high school students may have experienced heightened stress due to the upcoming national university entrance examination (gaokao), which may help explain their higher levels of depression and anxiety [49]. By contrast, our first-year undergraduates had already performed sufficiently well on the entrance examination to secure admission to a top-tier university. This may have contributed to their lower levels of depressive and anxiety symptoms. More generally, students admitted to top-tier universities in China may possess more effective coping strategies and problem-solving skills, which could buffer against psychopathological symptoms [50].

In our samples, male sex appeared to be associated with a higher risk of internet gaming disorder but a lower risk of IA, consistent with previous work in Chinese and US populations in which the 2 disorders were conceptualized as separate constructs [51,52]. Future research should examine whether and through what mechanisms sex influences the risk of either disorder, particularly given well-documented sex differences in internet use: male participants tend to engage more in computer gaming, whereas female participants tend to engage more in social networking and social media [53]. Previous studies have reported inconsistent findings regarding whether sex influences the risk of IGD or IA [54,55]. These inconsistencies may reflect differences in the relative proportions of individuals with IGD only, IA only, or comorbid IGD and IA.

### Limitations

Our findings should be interpreted with caution in light of several limitations. First, the cross-sectional design of this study precludes causal inference. Second, all data were obtained from self-report questionnaires, which may increase the risk of social desirability and recall biases. Third, the sample comprised exclusively Chinese participants residing in China, which may limit the generalizability of our findings to other cultural and geographic contexts. In addition, the inpatient sample size was relatively small, and many participants scored near the maximum possible values on several symptom scales, a phenomenon known as the ceiling effect. These factors may have reduced statistical power and, together with other sample-specific characteristics (eg, differences in sex ratios across samples), may help explain why several associations observed in the university and high school samples did not reach statistical significance in the inpatient sample. Nevertheless, most analyses conducted among inpatients showed patterns similar to those observed in the other 2 samples.

It should also be noted that our findings reflect IGD as a global construct without differentiating between specific game genres (eg, real-time strategy, massively multiplayer online role-playing games, sports games, or first-person shooters). Given evidence that the prevalence and psychological correlates of IGD may vary by game genre [56,57], future studies should systematically characterize predominant game types and examine genre-specific associations with psychopathology. In addition, the measures of psychopathological symptoms included in the 3 samples were not entirely consistent across the 3 samples (eg, the absence of SCL-90 in S2 and ASRS in S1), due to considerations such as survey timing constraints and primary study objectives. This inconsistency may have reduced the comparability of certain results (eg, associations involving ASRS) across samples. However, the key findings—namely, that IGD and IA are distinct yet moderately correlated constructs, with IA more strongly associated with the severity of psychopathological symptoms—were robust and consistent across all 3 samples.

### Conclusions

This study provides additional evidence that IGD and IA represent distinct yet interrelated constructs and further demonstrates that IA consistently exhibits a stronger association with the severity of psychopathological symptoms than IGD. These findings underscore the importance of recognizing and addressing compulsive and problematic online behaviors that extend beyond gaming, contributing to ongoing debates regarding the classification and clinical significance of behavioral addictions related to internet use, and highlighting the need for further refinement of diagnostic frameworks and the prioritization of targeted, evidence-based clinical interventions.

## Abbreviations

ASRS: Adult ADHD Self-Report Scale
*DSM-5*: *Diagnostic and Statistical Manual of Mental Disorders, Fifth Edition*
GAD-7: 7-item Generalized Anxiety Disorder
IA: internet addiction
IAT-20: 20-item Young’s Internet Addiction Test
*ICD-11*: *International Classification of Diseases, 11th Revision*
IGD: internet gaming disorder
IGDS9-SF: Internet Gaming Disorder Scale–Short Form
OR: odds ratio
PHQ-9: 9-item Patient Health Questionnaire
S1: sample 1
S2: sample 2
S3: sample 3
ZRE: standardized residual
